# Nano-Physical Characterization of Chemical Vapor Deposition-Grown Monolayer Graphene for High Performance Electrode: Raman, Surface-Enhanced Raman Spectroscopy, and Electrostatic Force Microscopy Studies

**DOI:** 10.3390/nano11112839

**Published:** 2021-10-25

**Authors:** Won-Hwa Park

**Affiliations:** Department of Chemistry, Hanyang University, Seoul 04763, Korea; s952151@gmail.com

**Keywords:** chemical vapor deposition, suspended graphene, Raman and surface-enhanced Raman spectroscopy, Radial Breathing Like Mode, electrostatic force microscopy, graphene ripple

## Abstract

To achieve high-quality chemical vapor deposition of monolayer graphene electrodes (CVD-MG), appropriate characterization at each fabrication step is essential. In this article, (1) Raman spectroscopy/microscopy are employed to unravel the contact effect between the CVD-MG and Cu foil in suspended/supported formation. (2) The Surface-Enhanced Raman spectroscopy (SERS) system is described, unveiling the presence of a z-directional radial breathing-like mode (RBLM) around 150 cm^−1^, which matches the Raman shift of the radial breathing mode (RBM) from single-walled carbon nanotubes (SWCNTs) around 150 cm^−1^. This result indicates the CVD-MG located between the Au NPs and Au film is not flat but comprises heterogeneous protrusions of some domains along the z-axis. Consequently, the degree of carrier mobility can be influenced, as the protruding domains result in lower carrier mobility due to flexural phonon–electron scattering. A strongly enhanced G-peak domain, ascribed to the presence of scrolled graphene nanoribbons (sGNRs), was observed, and there remains the possibility for the fabrication of sGNRs as sources of open bandgap devices. (3) Electrostatic force microscopy (EFM) is used for the measurement of surface charge distribution of graphene at the nanoscale and is crucial in substantiating the electrical performance of CVD-MG, which was influenced by the surface structure of the Cu foil. The ripple (RP) structures were determined using EFM correlated with Raman spectroscopy, exhibiting a higher tapping amplitude which was observed with structurally stable and hydrophobic RPs with a threading type than surrounding RPs. (4) To reduce the RP density and height, a plausible fabrication could be developed that controls the electrical properties of the CVD-MG by tuning the cooling rate.

## 1. Introduction

Graphene is a two-dimensional hexagonal nanostructure of carbon which exhibits excellent structural and electrical characteristics. They are extensively studied for their significantly improved mobility of charge carriers [1]. Noticeable development in the understanding of the properties of graphene is attributable to the study of graphene flakes, which are removed from bulk graphite by the application of mechanical force [1,2]. The exfoliated graphene flakes are small (<100 μm^2^), so while they are appropriate for the investigation of the basic characteristics of graphene, they are not suitable for the assessment of commercially viable large scale graphene fabricated by chemical vapor deposition (CVD) on Cu substrates using CH_4_ and H_2_ [3,4]. In particular, the precise analysis and optimization of the graphene properties suitable for wafer-scale dimensions are required for the evaluation and development of chemical vapor deposition monolayer graphene (CVD-MG) to further their applications.

With this in mind, (1) first, the blue-shift of the in-plane Raman G and 2D peaks of a CVD-MG on a relatively even Cu substrate domain (or nano-valley) with nearly uniform roughness are investigated and compared to the uneven case. A mechanical compressive strain is noticed to occur between the CVD-MG and the uniform Cu surface domain which is absent in the uneven (or nano-peak) case. Additionally, relaxing the compressive strain to reduce the agitation to the CVD-MG from the Cu foil substrate owing to the irregular Cu domains plays a crucial part towards the formation of suspended CVD-MG [5]. Thus, the influence of the compressive strain contributed by the structure of the substrate layer may be one of the cardinal keys in analyzing the performance (e.g., carrier mobility) of a CVD-MG electrode [6]. (2) Second, many investigations have been carried out to better interpret and exploit the broadly acknowledged contribution due to the electromagnetic (EM) field enhancement [7,8,9,10,11] and the chemical enhancement mechanism of CVD-MG which has been rarely studied [12,13,14,15,16,17]. Numerous studies have attributed the 4locally 5enhanced EM field, which occurs between noble metal nanoparticles (NPs), towards the generation of strong surface-enhanced Raman scattering (SERS) intensities. However, in the self-occurring agglomerates of nanoparticles, the SERS-active junctions or the hot spots occur at random positions. This results in a 5–6 orders of magnitude variation in the SERS intensity across hot spots even within identical specimens. Owing to the reproducibility of the SERS signal intensity, a nanoparticle-on-mirror (NPoM) system composed of Au NP/CVD-MG/Au films could be employed to comparatively explore the contribution of both in-plane and out-of-plane vibration modes at individual NPoMs according to the SERS intensity ratio [18,19,20,21,22,23,24,25,26]. It is worthwhile to note the significance of previous work by Park and coworkers pertaining to utilization of the Au NP/molecule/Au Film system [17,18]. As the EM enhancement factor of the Au NP/molecule/Au film was within ~106 and exhibited a nearly uniform intensity distribution, the NPoM-SERS system was utilized in this article. Due to the dipole–dipole interaction between Au NPs and the Au film, the SERS signal revealed the presence of the radial breathing-like mode (RBLM) associated with the normal-to-surface direction (z-axis) from spaced CVD-MG in the NPoM system, which was present in the low frequency region along with three nano-scroll-type defect peaks [20,21]. The peak intensity and position of the transverse 1, 2, 3 peaks below 700 cm^−1^ were also useful indicators of the tilted z-directional CVD-MG protrusion. Electrical characteristics of CVD-MG obtained by examining the degree of the tilted formation of out-of-pane modes is also useful in anticipating carrier mobility [24]. Moreover, it was shown that the z-polarized EM field was almost a factor of 50–70 times stronger at each NPoM compared to the xy-plane EM field, which significantly enhanced the I(RBLM)/I(2D) [20]. (3) To explore and make use of the key characteristics of CVD-MG, it is vital to study their chemical and atomic structures. A specific experimental method has been reported that explores both the nanoscale morphology of the CVD-MG and the electrical properties using electrostatic force microscopy (EFM) techniques [27,28,29,30,31,32,33]. Specifically, electrostatic force (amplitude- or phase change-) mapping can provide direct visualization of electric charge distributions associated with the threading or surrounded ripple (RP) patterns observed in CVD-MG on a SiO_2_/Si substrate using atomic force microscopy (AFM)-correlated Raman microscopy [34]. In particular, EFM-based investigations indicate that the threading-type RPs in CVD-MG produced a higher EFM amplitude, implying that threaded domain RP shapes are rather more equitable with fewer sp^3^-type defects than the surrounded domain RP shapes. (4) In addition, a novel method for increasing the electrical performance of large-scale CVD-MG by the control of RP density and heights subsequent to the transfer onto SiO_2_/Si substrates (or Au substrates) was achieved by varying the rate of cooling during fabrication [35]. It was found that CVD-MG fabricated with a high cooling rate demonstrated decreased RP densities and heights, which led to an improvement in the electrical properties such as charge carrier mobility and also reduction in the sheet resistance. The Raman studies conducted on these samples also showed a remarkable reduction in the article concentration of defects at higher cooling rates. Thus, a new method to achieve lower RP heights and density to decrease flexural phonon–electron scattering has been developed, which should lead to higher lateral carrier mobility [35]. Overall, this article systematically presents various characterization methods capable of detecting obstacles at each step of the fabrication process such that defect-free CVD-MG can be achieved, facilitating the improvement of large, transparent, and flexible electrodes with high electrical quality.

## 2. Materials and Methods

### 2.1. Graphene Synthesis on Cu and Transfer onto SiO_2_/Si Substrate

Graphene films were fabricated as follows. A roll of Cu foil was placed within a quartz tube furnace and subjected to a high temperature of 1000 °C and low pressure of ~10^−3^ Torr and annealed during ~20 min. The main purpose of the heat treatment is to increase the grain size which would aid in obtaining high-quality graphene films, as proposed by Li and coworkers [3]. Graphene was grown by the CVD process at 1000 °C and 500 mTorr with a CH_4_ (99.999%) flow rate of 50 s.c.c.m for over 30 min. Firstly, the sample assembly was quenched to 600 °C within ~5 min and then slowly cooled to room temperature ~55 min at low pressure of ~10^−3^ Torr. The as-grown graphene film was then mounted on a thermal release tape by employing slight pressure between two rollers. A bath filled with Cu etchant was utilized to remove the Cu substrate and the film was rinsed with deionized (DI) water. The CVD-MG on the thermal release tape was inserted between the rollers along with a target substrate, and exposed to a mild temperature of about 90~120 °C which facilitates the transfer of the CVD-MG from the tape to the target substrate. Specifically, the cooling rate was adjusted by precisely controlling the extent to which the furnace door of a vertical type CVD chamber was open throughout the cooling step. The XRD analysis of Cu foil, subsequent to an annealing treatment at 1000 °C, confirmed the presence of (0 0 1) preferred orientation. The sheet resistance of the CVD-MG was found by utilizing the four-point probe using a nano-voltmeter. For fabricating the SERS structure, a 10 nm thick gold film was deposited on a clean glass substrate using Au sputtering. The independent atomic force microscopy (AFM) studies determined the average root mean square roughness of the target Au surface to be about 0.3 nm. The gold coated substrate was used as a CVD-MG substrate for SERS experiment and A drop of Au colloidal nanoparticle solution with ~200 nm diameter was deposited on the fabricated CVD-MG/Au film and was air dried at room temperature [18,19].

### 2.2. Optical Characterization of CVD-MG on Cu and SiO_2_/Si Substrate

Optical characterization of CVD-MG by upright-type Raman spectroscopy is extensively used as a conventional method to understand the degree of doping [35], number of layers [36], and the influence of mechanical strain [6,34,35,37] predominantly employing in-plane phonon vibrations. Park and colleagues readily recognized the z-directional out-of-plane phonon vibrational modes, such as the RBLM and transverse nano-scroll-type defect modes of CVD-MG at each of the Au NP/Au film junctions via the SERS signal obtained using linearly polarized HeNe laser (633 nm) irradiation [20,21,25,26]. Generally, a 633 nm laser beam is utilized as the excitation source since it closely matches the localized surface plasmon (LSP) resonance of Au NPs and the coupled resonance surface plasmon polariton (SPP) of the Au NP–Au film junction system [18,19,20,21,22,23,24,25,26]. Based on these studies, the un-polarized SERS signals identified along the z-axis were attributed to the xy-plane axially symmetric SERS signal distribution from a z-directional dipole emitter (corresponding to the Au NP–Au film coupling orientation). A 532 nm laser was also applied for investigating the luminescent properties of CVD-CM on a Cu substrate.

### 2.3. Electrostatic Force Microscopy (EFM) Measurement

EFM measurements were performed using an advanced AFM system under ambient conditions. Amplitude modulation mode was utilized to explore the regional electrostatic interactivity amidst the tapping Au-coated tip and a bare PET (polyethylene terephthalate) surface as well as CVD-MG on PET. Please see the previous references for the detailed principle of EFM and how to get and understand the meaning of EFM image. Briefly, the two-pass scan procedure utilized in traditional EFM necessarily induces crosstalk between the AFM contours and the corresponding EFM image, irrespective of the lift gap applied for the subsequent scan (linear regime). The two-pass scan procedure heavily restricts not only the spatial resolution of the surface potential map but also the EFM signals. On the contrary, the EFM procedure used in this study was designed as an effective uni-pass scan procedure to simultaneously obtain both the AFM topography and the EFM image signal without compromising sensitivity. Moreover, the EFM procedure enables a thorough disconnection of the AFM contour and EFM image signals, since the individual signals were received at different specific frequencies. In particular, an amplitude modulation method was used to operate the EFM system using the two detection channels. As the surface of the sample displayed electrical properties, the interaction via the electrostatic force amidst the biased Au-coated tip and the sample was used to obtain the EFM image, while the van der Waals force between them was utilized to simultaneously obtain the AFM topography [30,31,32].

## 3. Applications and Characteristic Interests

### 3.1. Mechanical Strain Effect by Raman Spectroscopy and Microscopy of CVD-MG on Cu and SiO_2_/Si Substrate

In order to develop a mature technology based on graphene and to support its numerous applications, rapid characterization procedure and optimized control on the properties of largescale wafer-sized area CVD-MG is crucial. Nowadays, cardinal information on the thickness, substrate morphological effect, the extent of defect and the distribution of strain can be evaluated through Raman spectroscopy, in most cases. CVD-MG shows defect-induced Raman peaks at around 1360 cm−1 (D peak), and an overtone peak of nearly 2700 cm−1 (2D peak) [38,39]. These signals correspond to resonant Raman scattering where the laser excitation energy controls the resonant frequency and in turn the Raman shift [37,40,41]. It was reported that the variance in the gap between the positions of 2D and G band Raman peaks of CVD-MG mounted on the Cu foil was critical in the precise computation of the mechanical strain effect induced between Cu foil substrate and CVD-MG [37,42,43]. Subsequently, the Rayleigh scattering image, which provides the coarse topological shape of CVD-MG on Cu foil substrate, is obtained by using the Rayleigh scattered irradiation of 532 nm in space. This could be first utilized to provide and corroborate with mechanically strained distribution. It hinted that less mechanical association occurs between the Cu foil substrate and a CVD-MG at the uneven Cu domains (or PET and Au film substrate) which played a crucial role in the formation of suspended CVD-MG [6].

**Figure 1:**
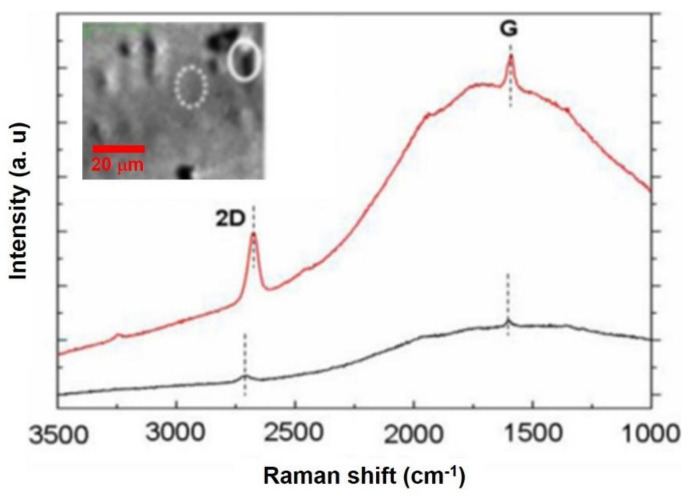
Raman spectra of CVD-MG deposited on Cu foil substrate. Inset showed the bright-field optical microscopy image of Cu foil substrate subsequent to the complete growth of CVD-MG. The image shows partial distribution of irregular Cu domains in the micrometer range. The Raman spectra denoted by the red and black plots were obtained from the solid and dotted circle regions of the inset image. Reprinted with permission from ref. [6].

In Figure 1, the conventional Raman spectra of a graphene on the Cu surface were exhibited. Due to single, sharp, and symmetric 2D peak shape at both the solid and dotted regions of the optical image, it could be speculated that the CVD-MG was monolayer and threaded at the solid circle region. Actually, it was too vague to estimate the concerns occurring due to defects when we applied a 532 nm excitation light source to CVD-MG on Cu substrate. Due to a high intensity emission from Cu substrate, the defect signal (D-band) might not be visible. It was important to note the full width at half maxima (FWHM) of G-band increased violently with the concentration of defects in comparison with a FWHM of natural graphene of G-band (13~15 cm^−1^) [39,44,45,46,47]. This might reveal that the defect concentration in CVD-MG was higher than in the pristine and, as discussed previously, there might be a hidden D-band peak in the luminescence backdrop of Cu. However, if the extent of defect influence is less significant than the collision of ions, there might be no significant occurrence of D peak [38]. Moreover, it was known that the positions of G and 2D band peak did not evidently switch, with gaining defects estimated from raising the ionic concentration. It implied that defect affection might be so small in investigating the shift in the peak of CVD-MG regarding mechanical strain, which was dependent on substrate morphology [6,43,48]. Although it is so marginal, the effect of differently strong coupling of the CVD-MG to the Cu surface (red and black spectrum) owing to possibly different orientation of neighboring Cu grains or slight traces of Cu surface oxidation (CuO_2_) may be attributable to explain slightly different band positions and varying band intensities.

**Figure 2:**
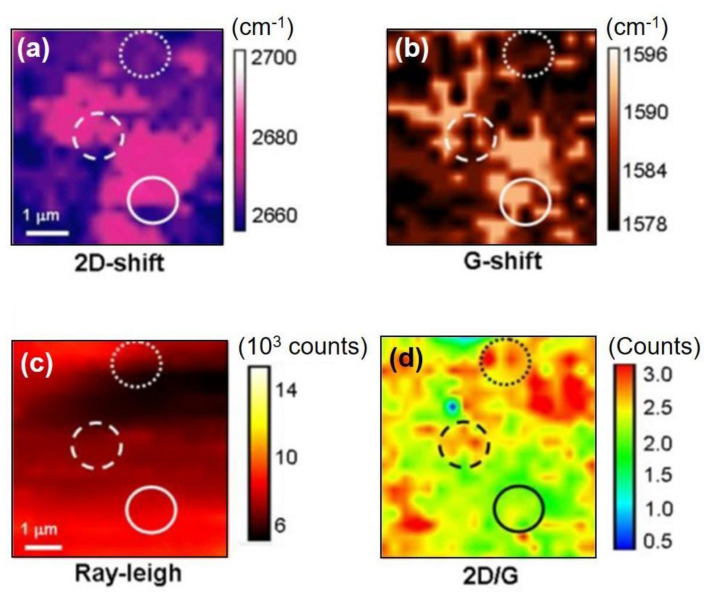
(**a**,**b**) were the 2D and G peak shift Raman images, respectively. Dotted and solid circle areas in each figure represented the regions where the largest variation in the shift was generated. (**c**,**d**) were the Rayleigh scattering and graphene thickness (2D/G) images, respectively. All images were reprinted with permission from ref. [6].

**Figure 3:**
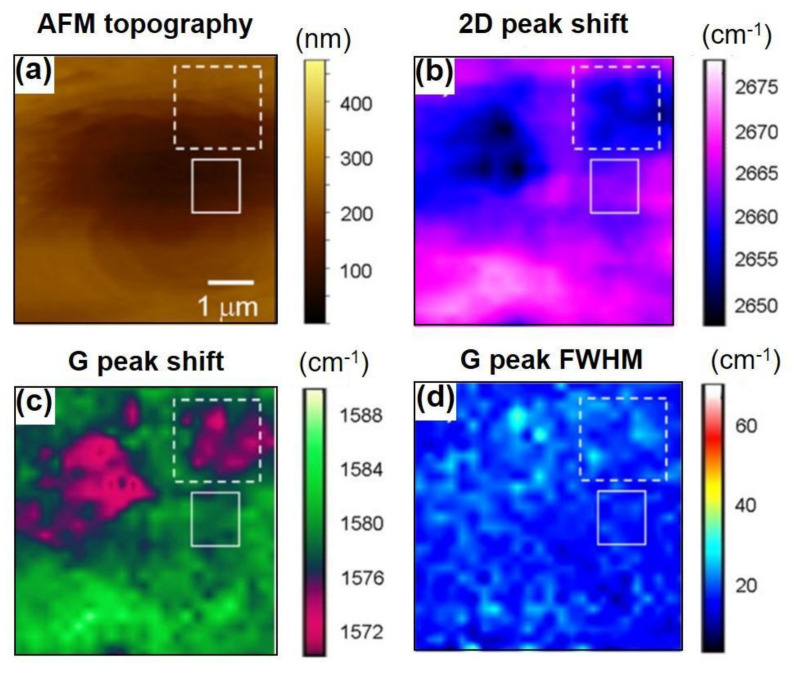
(**a**) AFM topography of monolayer on Cu substrate. (**b**–**d**) were the corresponding Raman images of 2D, G peak shift and G peak-FWHM (G peak Full-Width @ Half Maximum). Dashed square regions in each image indicated that the irregular fixture from AFM topography played a cardinal role to red-shift the 2D and G peak location. It could be compared to the solid square region between 2D- and G-peak shift, where the 2D peak was stronger blue-shifted than the G-shift, meaning that mechanical strain effect is more influential. The doping effect of deposited CVD-MG from underlying Cu substrate was not evident in terms of both stronger 2D peak shift than the G peak case and the vague relationship between G-shift and G-FWHM. Reprinted with permission from Ref. [43].

Moreover, Raman spectroscopy/microscopy technique has been utilized so as to unravel the direct contact effect between CVD-MG and Cu foil substrate in terms of suspending or supporting formation of CVD-MG eventually on the basis of mechanical strain phenomena revealed by Raman peak shift. In other words, reducing the compressive strain to minimize the agitation from Cu foil substrate to the CVD-MG, owing to the uneven Cu domain playing a crucial role towards the formation of suspended CVD-MG. As such, the effect of mechanical strain caused due to the underlying substrate structure could be proposed as one of the cardinal keys in determining superior performance (ex: carrier mobility) of CVD-MG considering suspended shape, as investigated in Figure 2 and Figure 3 [6,43]. The detailed quantitative comparison between dotted and solid circle is summarized in Table 1.

**Figure 4:**
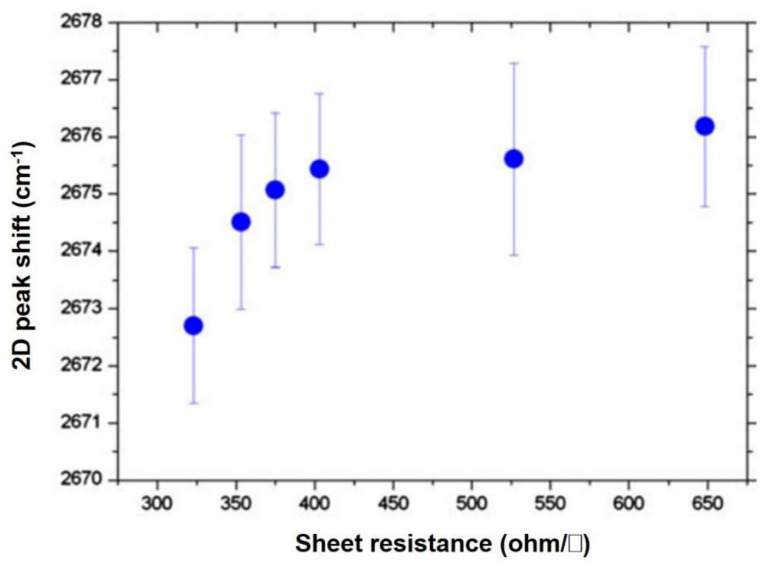
Specific relationship between shift in the 2D peak of CVD-MG on Cu surface and the value of sheet resistance computed after transfer onto the PET film. Reprinted with permission from ref. [43].

**Figure 5:**
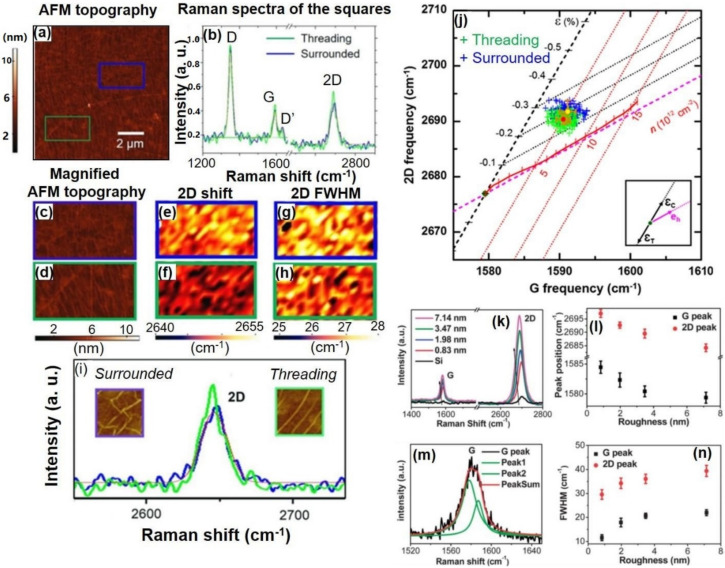
(**a**) The AFM image of CVD-MG on Au film was shown. The regions marked with blue and green squares clearly revealed different RP shapes such as the surrounded and (**c**,**d**) threading type, respectively. It is noticed that the blue and green squares were completely correlated in Figure 5, particularly the square boundary color in (**c**–**i**) including spectra color. (**b**) The representative Raman spectra at each colored domain were shown. The blue and green Raman spectra correspond to the surrounded and threading regions, respectively, Lorentzian curve fitting shown as red color, with focus on blue (threading) domain and green (surrounded) in (**i**). The (**e**)/(**f**) and (**g**)/(**h**) images showed the correlative maximum peak position and FWHM of 2D mode Raman image for the different colored regions, respectively. It was clearly shown that the 2D FWHM of the green domain showed a smaller value than the blue, which indicates that the threading type ripples (RPs) displayed relatively stable structure. The inference is also supported by the lower FWHM value and the relative red-shift than that of the surrounded. (**i**) The representative Raman spectra of each RP shape of CVD-MG were displayed (blue and green were also the surrounded and threading type, respectively) and the corresponding RP shapes were also shown in the inset of (**i**) with the coincided color. Reprinted with permission from [33]. (**j**) Correlation graph for different types of RP on CVD-MG. Correlation between G and 2D modes of the surrounded ripple (blue symbols) and threading (green symbols) were shown. Inset: arrows marked as ε_C_, ε_T_, eh represent vectors from the zero point (ω_G0_, ω_2D0_) affected by compressive strain, tensile strain and hole doping, respectively. (**k**) Raman spectra of CVD-MG on the Ag thin films as a function of roughness. It showed evident red-shift of peak position for both G peak and 2D peak. (**m**) Split of the Raman G peak of graphene on the Ag film of roughness about 3.47 nm. (**l**) Positions of G and 2D peaks based on Ag film roughness. (**n**) Full width at half maximum (FWHM) of G and 2D peaks with respect to Ag film roughness. Reprinted with permission from ref. [48].

In consideration of the large area applications of CVD-MG with largescale, transparent, and flexible electrodes, investigating the electrical performance of CVD-MG after mounting onto the flexible plastic based substrate such as polyethylene terephthalate (PET) film was essential. Numerous factors would be considered in improving the electrical performance of CVD-MG on PET substrate. Interestingly, it could be revealed that the particular relationship between the magnitude of mechanical strain, expressed by the degree of 2D peak blue-shift, and sheet resistance, was revealed in Figure 4. Previous studies have shown that the suspended graphene film on shallow wells of diameter in the order of a few µm and 2 µm deep have shown reduced sheet resistance, which was in regard with the red-shifts of G peak, resulting in the significance of the formation of suspended CVD-MG in order to achieve improved electrical performance [49]. Likewise, Figure 4 exhibited the comparable relationship amidst the Raman 2D peak shifts and the correspondent sheet resistance value after mounting the CVD-MG onto the PET substrate. Considering that both G- and 2D-band peaks were all in-plane vibrational modes, it could be claimed that the tendency shown in Figure 4 was somewhat understandable. It was noticed that the variation in the 2D peak shift resulted in a proportional change in the sheet resistance value. Further studies are required to clearly comprehend the detailed mechanism, however, it could be inferred that one of the significant parameters towards the superior electrical properties might be found during the initial stages of graphene growth. Specifically, an increase in the concentration of suspended domains on the Cu surface would improve the electrical properties on PET substrate. This shall be achieved by retaining as many intact CVD-MG domains as possible by forming suspended CVD-MG during the rapid cooling step [41,50,51]. As a result, the suspended and intact domains of CVD-MG might have increased scope to get to non-agitated condition even after the wet-transfer process to PET. However, those of the relatively perturbed domains, due to the superior adhesive interaction amidst CVD-MG and Cu surfaces, may result in increased sheet resistance value on PET. The issue continues to be challenging, as does undertaking work to discover the accurate mechanism about the “memory” phenomenon of mechanical strain aspects from Cu to PET substrate transition. In addition, the corroborated AFM-Raman system could identify “threading” and “surrounded” RP types [34]. Figure 5a shows the AFM images corresponding to the varying RP shape domains, identified by the blue and green colored regions of a CVD-MG. The RPs are noticed to display an intense spread on the Au film substrate, and the corresponding Raman spectra are displayed in Figure 5b. The blue and green colored squares revealed in Figure 5a show with clarity the different RP shapes which are confirmed from Figure 5c,d as surrounded and threading types, respectively. It is noted that the blue and green square colors were precisely identified from Figure 5. The AFM images also show that the two square regions display disparate RP fixtures. The 2D Raman peak positions for these regions were not identical. The AFM topographic images, the images corresponding to the maximum peak position of 2D mode and 2D-FWHM Raman mode images for two square regions are sequentially displayed in Figure 5c–h. The higher FWHM of the 2D mode in the blue domain was spatially resolved and imaged with the corresponding higher 2D peak maximum position image, as shown in Figure 5e, g. The enlarged AFM topographic images reveal that the RPs in the blue domain were of the surrounded type, while those in the green region corresponded to the threading type. From this structural point of view, the employed typical Raman spectroscopic investigation by studying the shape of the 2D peak could also give significant insight in regard to the sample surface structural conditions from threading RPs in terms of relatively exerted mechanical strain distributions as unveiled by red-shift of 2D peaks with relatively weak asymmetric shape [34]. Groups by Ryu suggested a template to elucidate the correlation between strain and doping effects [52]. Then, to further investigate strain effects, the template by Ryu could be employed. Hundreds of spectrums are obtained from the mapping of blue and green squares in Figure 5a, and information corresponding to G and 2D peaks are extracted using lorentzian fitting. In Figure 5j, point distributions for both types were positioned to the compressive strain region with moving towards the p-doping vector, in which blue points as a surrounded type were affected by stronger strain (0.2%) than green points for a threading type (0.15%). That in (Figure 5k) showed Raman spectra of CVD-MG on the silver thin films with varying levels of roughness. An evident shift towards the red region for both G peak and 2D peak positions was noticed. (Figure 5m) The split in the Raman G peak of graphene on the Ag film with the roughness of 3.47 nm is observed. (Figure 5l) The position of G and 2D peaks with respect to the Ag film roughness is shown. (Figure 5n) Full width at half maximum (FWHM) of G and 2D peaks with varying roughness of the Ag film [48].

### 3.2. Surface-Enhanced Raman Spectroscopy (SERS) of CVD-MG with Nanoparticle-on Mirror (NPoM) System

The preparation of SERS-active metal nano-structures which provide predictable, reliable and intense Raman signals is extremely complex. This problem is still being overcome by methods such as by producing self-assembled nanoparticle–molecule monolayer–film junctions, called the Nanoparticle-on-Mirror (NPoM) system [18,19,20,21,22,23,24,25,26] that generates significantly reproducible SERS signals at the single-junction level. Moreover, exploring catalytic chemical reactions probed by plasmon-driven coupling has been done by Yoon and colleagues via NPoM system [53,54,55]. Furthermore, the unique metallic structure of NPoM, composed of Au NP/CVD-MG/Au film in this article, could be a good candidate in terms of studying out-of-plane vibrational modes thanks to strongly generated z-polarized electromagnetic (EM) field contribution in comparison with in-plane vibrational modes such as G- and 2D-modes [20,21]. As a result of the NPoM-SERS experiment, Radial Breathing Like Mode (RBLM) associated with surface-to-normal directional (z-axis) vibrational phonon modes from the spaced CVD-MG could be exclusively observed (or enhanced) by dipole–dipole interaction between Au NP and Au film in the low frequency region at ~150 cm^−1^ [22], which was quite similar to the Raman shift position of Radial Breathing Mode (RBM) from single-walled carbon nanotubes (SWCNTs) [55].

**Figure 6:**
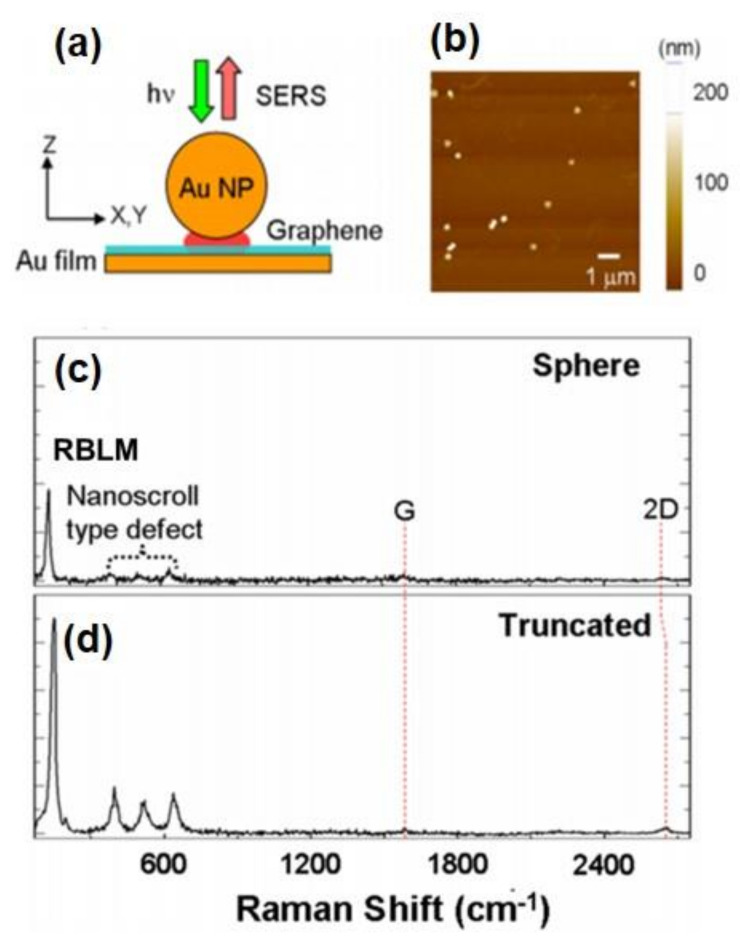
(**a**) Schematic picture for CVD-MG experiment by NPoM-SERS technique. (**b**) AFM image of the Au NP/CVD-MG/Au film system. (**c**,**d**) The typical SERS spectra were displayed at (**c**) spherical and (**d**) relatively truncated Au NPs. Reprinted with permission from ref. [20,21].

In this part, both xy-plane (in-plane) and upward z-directional (out-of-plane) phonon vibration of CVD-MG spaced between an Au NP and Au film junction are simultaneously used, which could make a pivotal contribution to the quantitative assessment of the role played by the z-polarized (or localized) electromagnetic (EM) field in this experiment. The RBLM mode, which was ascribed to the presence of a strongly enhanced z-localized EM field by virtue of dipole–dipole interaction between Au NP and Au film, could be newly appeared (or enhanced) [20,21,22]. In addition, this experiment showed the relative contribution between in-plane and out-of-plane vibrational mode in a quantitative way by employing I(RBLM)/I(2D) value at each junction; it could be shown that almost 50~70 multiples stronger z-localized EM contribution was found at these NPoMs in Figure 6a,b. Additionally, it could also be ascertained that an Au NP with a relatively truncated shape showed about 1.3 multiples higher z-polarized EM contribution than an Au NP which possessed a nearly spherical shape due to the increased concentration of the sharp and needle-like part from the curtailed Au NP in this work, as shown in Figure 6c. Moreover, it could be also explored that the out-of-plane vibrational phonon modes present at around the 300~700 cm^−1^ region, which was located next to higher frequency regions than strongly enhanced RBLM at 150 cm^−1^ by the same NPoM system. These 3 low frequency peaks could be called a transverse acoustic mode series (TA1, 2, 3), and utilized for efficiently and precisely investigating the tilted out-of-plane vibrational subtle motion of CVD-MG along the z-axis. However, the accompanied TA peaks between 300 and 700 cm^−1^ have not yet been investigated. At this juncture, the characteristic Raman behaviour of SWCNTs is worth a mention. In SWCNTs, a few of the identified Raman peaks displayed below 700 cm^−1^ and this could be indicated as an intra-valley scattering or could be attributed to the vibration of the radius of nanotubes [56]. Since the lower-frequency vibrations showed equivalence towards the translation of the honeycomb structure to a curvature [38], it was clear that the out-of-plane vibrational modes were mainly exhibited due to the relatively higher phonon frequency of SWCNTs. Specifically, the vibrational modes of SWCNTs which are less than 700 cm^−1^ could be analyzed with ease as out-of-plane phonon modes by considering the protruded CVD-MG on xy-plane at our NPoM. With references from the investigation of SWCNTs, TA peak series were analyzed with caution and it was revealed that the out-of-plane vibrational modes of a CVD-MG at the lowest frequency (TA1) showed a stronger out-of-plane vibration. However, this was mildly inclined towards the RBLM phonon direction. The TA2 and TA3 signals could be shown as components with relatively larger tilt adjacent to RBLM mode [25].

**Figure 7:**
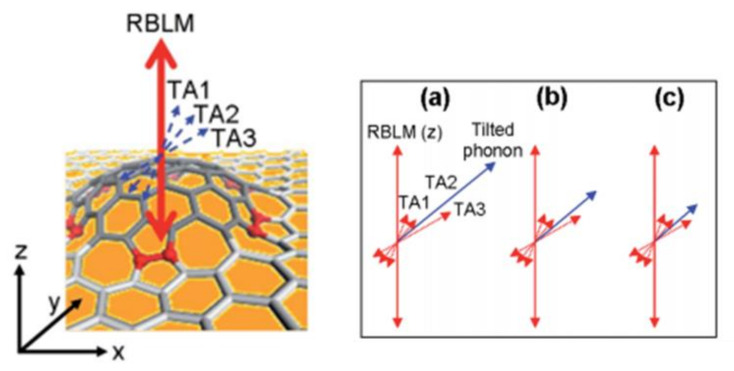
(**Left**) Illustration depicting the probable protruding conformation by combining TA series signals with strong RBLM. (**Right**) (a)~(c) Vectorial combination of tilted vibrational modes (blue) with RBLM vibrational mode (red) direction (tilted vibrational modes: TA1~TA3, dotted red arrow). TA3 signals with varying intensity led to the variation in the magnitude of the tilt formation of CVD-MG [25]. Reprinted with permission from ref. [25].

Under this point of view, it could be claimed again that CVD-MG was not always flat and some domains were heterogeneously protruded, especially upward along the z-axis, as shown in left Figure 7. Note that if uniaxial pressure toward the sample substrate (Au film) by loading of Au NP was exerted, it might not expect the appearance of z-directional upward out-of-plane vibrational modes along the z-axis from a CVD-MG. However, RBLM and other nano-scroll type defect peaks at <700 cm^−1^ should be evidently observed, meaning that uniaxial effect by loading of Au NP was marginal [54]. The precise structural analysis of CVD-MG spaced in Au NP-Au film junction could be one of the useful characterization formats in terms of establishing a correlation between the degree of CVD-MG roughness and macroscopic electrical performance of a CVD-MG [6,25].

**Figure 8:**
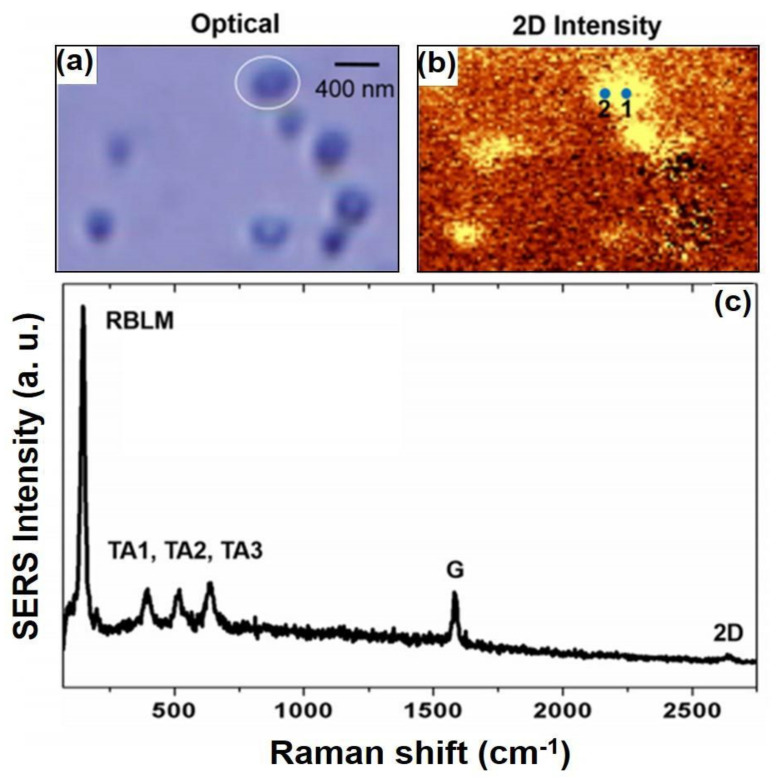
Bright-field and 2D intensity Raman images of CVD-MG at Au NP-Au film plasmonic junctions at the same region were shown in (**a**,**b**), respectively. The numbers in (**b**) indicated the location on the sample wherein the SERS spectra were recorded. (**c**) The representative SERS spectrum of an sGNR with immensely improved G band intensity was displayed. Reprinted with permission from ref. [61].

Graphene nanoribbon (GNR), a promising 1D carbon nanostructure, plays an important part for possible electronic applications. This attribute may be due to the lateral electron confinement in the ribbons which opens a band-gap in graphene. These elongated strips of graphene have recently attracted both theoretical and experimental studies due to their immense potential as basic building blocks in nano-devices and spin electronics [54,55,56,57]. This was reported by a study on scrolled graphene nanoribbon (sGNR) which were generated by means of CVD method by Park and co-workers, especially discovered by NPoM-SERS experiment in Figure 8a,b. The presence of sGNRs which occur between Au NP and the Au film junction system could be recognized by the high and exclusive increment in G peak coupled with a succeeding deconvolution and the resulting splitting of G peak (G+ and G−) with strong RBLM enhancement, as displayed in Figure 8c. More specifically, owing to the weak adhesive force between CVD-MG and target Au film during transfer might result in scarce distributions of CVD-MG, especially by a z-directional curvature-induced force and SERS spectral G peak splitting [58,59,60]. Figure 8a,b showed the optical (bright-field) and the corresponding 2D intensity SERS image of CVD-MG, respectively. Following previous works, RBLM at ~150 cm−1 and 3 TA mode series at 300~700 cm−1 could be explored. The matching 2D intensity SERS image revealed a non-homogeneous spread of CVD-MG due to the weak force of adhesion between CVD-MG and Au film as compared to SiO2/Si, resulting in highly deficient distribution with scrolled shape and resultant weak intensities of Raman 2D signals. This property might result in scrolling CVD-MG on Au film. However, this phenomenon was not observed to be homogeneous. Further, there were no predominant enhancements of G mode. Nevertheless, it could be claimed that a sGNR was observed at certain junctions. Moreover, some bright spots were noticed with clarity at the Au NP-Au film junction, and the correspondent SERS peak intensity of 2D peak (or G peak) could be enhanced.

**Figure 9:**
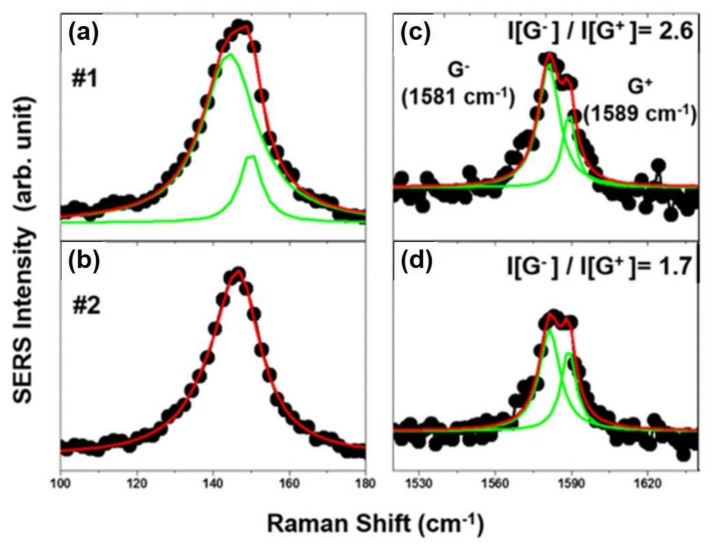
(**a**)/(**c**) and (**b**)/(**d**) showed the corresponding Raman peak shapes of RBLM and G mode for #1 and #2 positions exhibited in Figure 8b, respectively. Immensely increased G peak could be decoupled into G^−^ and G^+^ with each intensity ratio written at the upper-right locations in (**c**,**d**). The calculated higher I(G−)/I(G+) values were corroborated with the higher RBLM intensity and higher asymmetric RBLM peak shape. Reprinted with permission from ref. [60].

It could be observed the SERS spectrum of a CVD-MG at #1 position in Figure 8b and displayed in Figure 8c with highly and exclusively enhanced G peak intensity. For more precise analysis, the G peak was re-examined and it was figured out that the G peak could be de-convoluted into G^−^ (1581 cm^−1^) and G^+^ (1589 cm^−1^). This separation could be attributed to the curvature in the graphene ribbon which lifted the degeneracy between the LO and TO phonons near the Γ-point of the graphene Brillouin zone. This has resulted in a separation of the G band into decomposed peaks placed at both 1581 and 1589 cm^−1^ by z-directional strain effect. The intensity ratio between G^−^ and G^+^ is quantitative evidence to obtain the degree of scroll formation of CVD-MG, as shown in Figure 9. Owing to the relatively weak force of adhesion between CVD-MG and Au film during transfer, the CVD-MG mounted on Au film might show limited distribution and the larger extent of scrolling which would result in CVD-MG of much smaller size possessing curvature fragments such as sGNR.

### 3.3. Employment of Electrostatic Force Microscope (EFM) Technique

In order to better understand the scalability and flexibility of transparent graphene-based electrodes, the local electrostatic force distribution of the PET film surface before and after transferring CVD-MG was investigated. This aided in understanding the influence of local electrostatic interaction between CVD-MG and PET substrate. So that the measurement of sheet resistance value of deposited CVD-MG on PET is always indispensable, the electrical modulation of amplitude was used in a tapping mode AFM tip to obtain the distinguished electrostatic force amplitude mapping of CVD-MG [27,28,29,30,31,32,33]. This assisted in determining the various electrostatic attractive interactions that were present between Au-coated tip and bare PET. The observed difference could influence the formation of graphene and its electrical characteristics, which are correlated to the difference of sheet resistance value. More specifically, scanning probe microscope (SPM) methods are emerging in recent years as a useful tool for unraveling the microscopic (or nanoscopic) local surface morphology via atomic force microscope (AFM) and local electrical properties such as conductance mapping using a conductive atomic force microscope (C-AFM) in regard to local electrical properties and their corresponding electrical macroscopic properties [62]. The shape of Au-sputtered nano-gap could be also visualized through measurement of the varying EFM tapping phase as a function of employed DC electrical bias voltage [31]. Recently, A. C.-Gomez and colleagues investigated the point of subsurface charged impurities on the local electronic characteristics of graphene extracted by mechanical exfoliation on silicone oxide, employing the EFM technique. They highlighted that the “perturbations of the carrier density at the microscopic level remarkably changes the electronic properties of CVD-MG, thus the characterization of local charge distribution on CVD-MG and its subsurface was fundamental for enhancing the performance of graphene-based devices,” [30]. In this regard, approaches were made to characterize the electrostatic force mapping of plain PET film and the corresponding CVD-MG on the PET film systems. Utilizing the amplitude demodulation EFM method, it could be precisely explored target substrate charge distribution and resultant the local electrical performance. So the interdependence of bare PET and CVD-MG on PET on the magnitude of electrostatic force interaction could be eventually revealed. In addition, the final relation of the magnitude of electrostatic interaction between CVD-MG and PET surfaces could be compared with the value of macroscopic sheet resistance.

**Figure 10:**
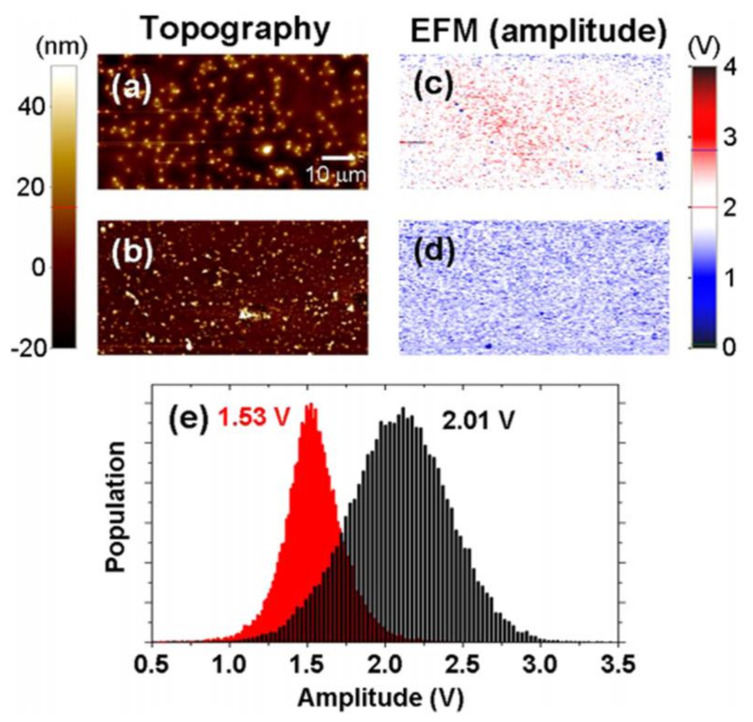
(**a**,**b**) were the topographies of the plain PET substrates. Nanoscale fixtures of silicone adhesive showed a uniform distribution. (**c**,**d**) were the corresponding EFM amplitude images of (**a**,**b**), respectively. (**a**) exhibited higher tapping amplitude (less electrostatic attractive) distribution than the (**b**). The distribution of tapping amplitude which is caused due to the electrostatic force between bare PET substrate and Au-coated AFM tip was shown in (**e**). Reprinted with permission from ref. [30].

**Figure 11:**
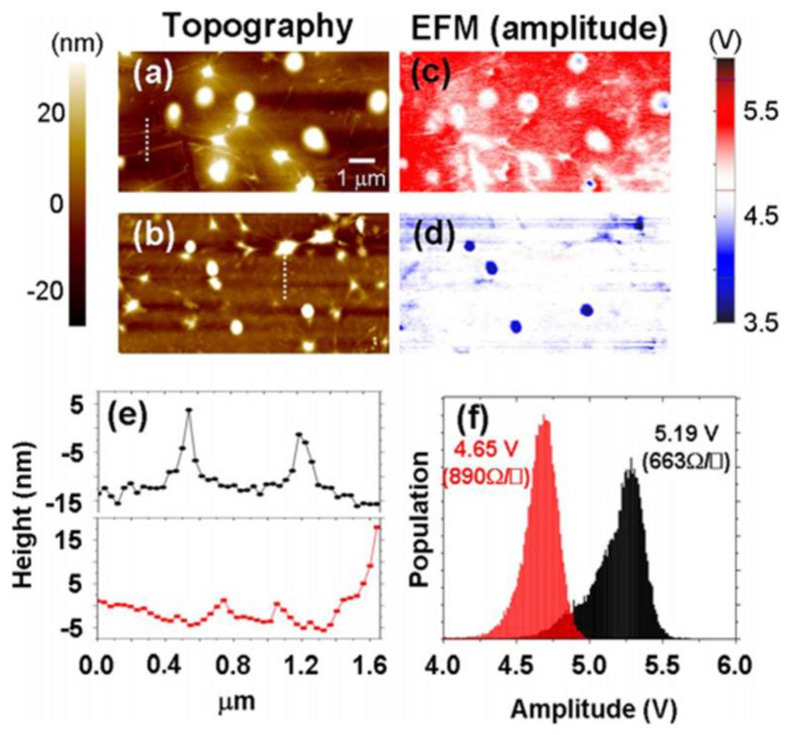
(**a**,**b**) showed the contour of the transferred CVD-MG on PET substrate. The CVD-MG boundaries in (**a**) were evidently noticed, while those in (**b**) were hardly seen due to stretched formation of CVD-MG boundaries toward the PET surface. (**c**,**d**) were the corresponding EFM amplitude images of (**a**,**b**), respectively. (**e**) Dotted line profiles of each topography were utilized to clearly identify the boundary shape of the transferred CVD-MG films. The upper line profile showed the case of (**a**) and the bottom profile indicated (**b**) case. (**f**) Statistical distribution of electrostatic amplitude of each case. Red and black histogram indicating the (**c**,**d**) cases, respectively. The red histogram shows the strong electrostatic attractive force with PET surface and the correlating higher sheet resistance value, whereas the weak electrostatic attractive force with PET surface and correlating lower sheet resistance value are presented in the black histogram. Reprinted with permission from ref. [30].

Figure 10a,b showed the AFM topography images corresponding to the surface of the two types of bare PET films. The nanoscale regular fixtures derived from silicone adhesive were vividly identified. Figure 10c,d reveal the corresponding EFM amplitude images. Interestingly, the relatively higher electrostatic force amplitude distribution of PET displayed in Figure 10c was noted in Figure 10a, while the lower value distribution of Figure 10d was noticed in Figure 10b. Alternatively, Figure 10a displays higher electrical-based tapping amplitude, where the interactivity of the weak electrostatic attraction progressed relatively. On the contrary, a feeble tapping amplitude was generated due to a relatively stronger attractive interaction as observed from Figure 10b. The insulating nature of PET film is confirmed by the electrostatic force distribution shown in Figure 10e. We initially considered that bare PET film would fail to show electrostatic force variation in space sustaining normal set point amplitude. Our belief was supported by the absence of sheet resistance when the measurement was conducted by a macroscopic method. However, a significantly different electrostatic force distribution was noticed between the bare PET films (2.01 V/1.53 V) as shown in Figure 10a,b. This could be attributed to the presence of some defect regions where the local unbalanced charged areas of bare PET surface were distributed, which failed to be identified by macroscopic sheet resistance. On the basis of results on the bare PET experiment using EFM technique, transferred and charged CVD-MG might hold a high expectation of introducing tapping amplitude variation. As such, we attempted to utilize the EFM method to analyze the CVD-MG on a PET substrate system corresponding to the local electrostatic behavior in regard with the availability as a flexible CVD-MG substrate with high electrical performance. Figure 11a,b revealed the topography images of CVD-MG on a PET substrate. The corresponding EFM amplitude images were presented in Figure 11c,d with 10 μm × 10 μm dimensions. To our interest, the topography of CVD-MG boundaries and some edge regions on PET substrate were distinctly seen, and the top region of Figure 11e reveals the corresponding line profiles. However, by contrast, the graphene boundaries were not clearly visible in Figure 11c,d. The graphene sheet and boundaries constituted a “graphene-tent” since they appeared to be stretched towards the PET surface [30].

All the EFM amplitude images and the resulting distribution showed coincidence with the amplitude distribution obtained previously for bare PET. Figure 11a presents the amplitude of bare PET film as 2.01 V, which maps well with the higher electrostatic amplitude (5.19 V) and lower sheet resistance value (663 Ω/□). In contrast, the amplitude of bare PET film shown in Figure 11b was 1.53 V, which matched with the lower electrostatic amplitude (4.65 V) and relatively higher sheet resistance value (890 Ω/□). Irrespective of the system being considered as bare PET or a CVD-MG on PET substrate, a higher electrostatic tapping amplitude was recorded. This showed that the tip-sample surface electrostatic interaction was not quite attractive, resulting in a less perturbed system in electrical performance of the CVD-MG upon PET surface. However, the lower electrostatic tapping amplitude distribution led to a highly perturbed system between the CVD-MG and the underlying PET surface. This indicated a strong electrostatic interaction between the tip and sample surface [30]. In addition, employing EFM technique, comparative studies on the hydrophilic and hydrophobic properties which are dependent on RP shape such as the threading RPs and surrounded RPs have been performed [34]. In particular, EFM was a dynamic non-contact AFM type where the electrostatic force was investigated via tip tapping modulation (amplitude or phase shift) with sample surface. In most of the cases while using EFM, the surface properties of the sample would be obtained from electrical properties. The interaction force would act as the electrostatic force between the biased Au-coated tip and sample. In particular, electrical properties of samples could be obtained based on the charged/hydrophilic nature of the sample while displaying unbalanced charge population (ionic) or no-charged/hydrophobic nature while expressing balanced charge population, such as alkyl group-dominant specimen (non-ionic). For example, if the tip was positively charged and the sample was grounded, the tapping amplitude of the tip could be modulated based on the surface charge of the sample. The path traced by the cantilever indicated that the EFM image could be obtained in accordance with the relative electrostatic attractive or repulsive force.

**Figure 12:**
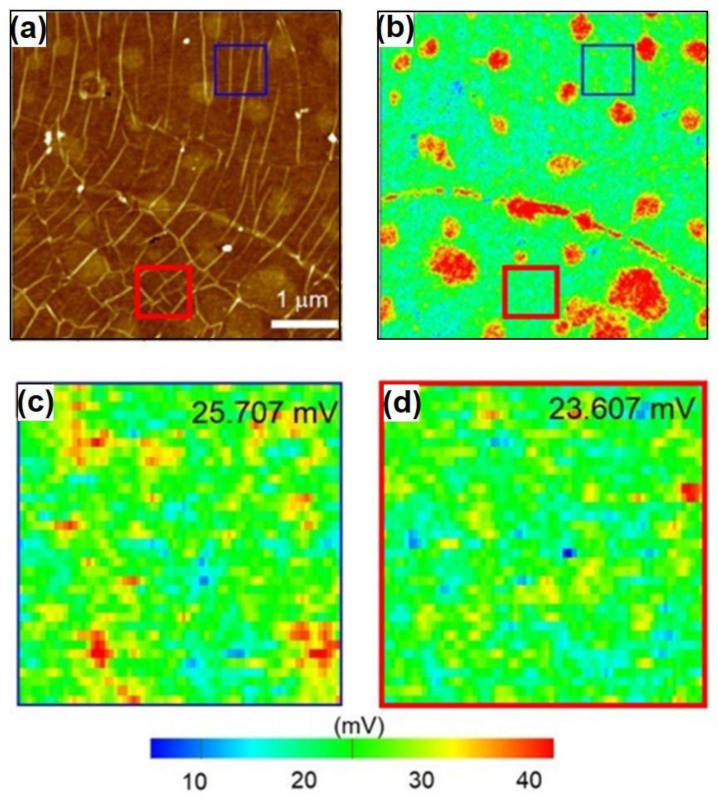
(**a**) The AFM image revealed both the threading (blue square) and surrounded type RPs (red square). (**b**) The corresponding EFM image simultaneously obtained with AFM. Silicone adhesive regions in (**b**) were not considered. The (**c**,**d**) were zoomed-in EFM images at each chosen region with averaged EFM tapping amplitude voltage values written in upper-right positions. Reprinted with permission from ref. [34].

Figure 12a showed the AFM topography of CVD-MG transferred on SiO_2_/Si substrate that included both threading (blue square) and surrounded (red square) RPs, and Figure 12b exhibited the corresponding EFM taping amplitude images. The regions marked in red with a high EFM amplitude voltage may correspond to the residual domains in PMMA. Since PMMA is an established hydrophobic polymer, the electrostatic oscillating amplitude between an Au-coated tip and PMMA surface could be large, leading to a region with high EFM voltage. We exclusively choose the regions (the blue and red square in Figure 12b–d showed the magnified CVD-MG domains to avoid PMMA residual domains. The averaged amplitude voltages were exhibited on the top-right location of each image in Figure 12c,d. As a result of EFM, the CVD-MG domain with threading-type RPs (blue square) showed a higher amplitude voltage than the surrounded (red square). Practically, an absolute graphene must remain in its native form, namely, free-standing or suspended type, and maintain very high hydrophobicity. It shows no disturbance in terms of sustaining and lateral transporting carrier sources perfectly, and shall be addressed as the charge neutral status. Nevertheless, non-desired influences such as abnormal doping or strain effect might occur which may result in the opening of the bandgap at Dirac point leading to an unbalanced/disordered charge status. The relatively higher EFM tapping amplitude voltage distribution indicated in the blue square than that of the red showed that the threading RP domains were relatively charge balanced (or nearly neutral) regions and anticipated relatively higher carrier pathway domains as well.

### 3.4. Enhancement of Electrical Property by Controlling Cooling Rate during CVD Process

A novel method to improve the electrical performances of large area CVD-MG via governing the RP concentration and height after mounting onto the SiO2/Si substrates by varying the rate of cooling during CVD was demonstrated [35,36,40,41]. Although numerous challenges in CVD-MG products still persist, such as non-uniform distribution of defects of various kinds and volatile dopants, they were removed with ease during the reliability test, which led to a decrease in the value of sheet resistance value. Other alternative methods such as hybridizing a CVD-MG with Ag nanowires have been investigated. Combining a 2D CVD-MG with 1D Ag nanowires in a hybrid film holds the potential to improve the electrical properties such as lower sheet resistance value. It is also robust against electrical breakdown, with insignificant decay of the optical transmittance [61]. In order to improve the electrical properties of CVD-MG, Park and coworkers have suggested that the variation in the rate of cooling and the concentration of hydrocarbons in the cooling phase has resulted in graphene islands with different degrees of sizes, nuclear densities, and growth rates. The density at the nucleation site on a Cu foil surface was drastically reduced during a rapid cooling procedure. Thus, introducing a novel fabrication method to improve the electrical properties of CVD-MG on the Cu surface could be studied with respect to the details of deformed nano-physical shapes and RPs of CVD-MG on SiO2/Si substrates after transfer.

**Figure 13:**
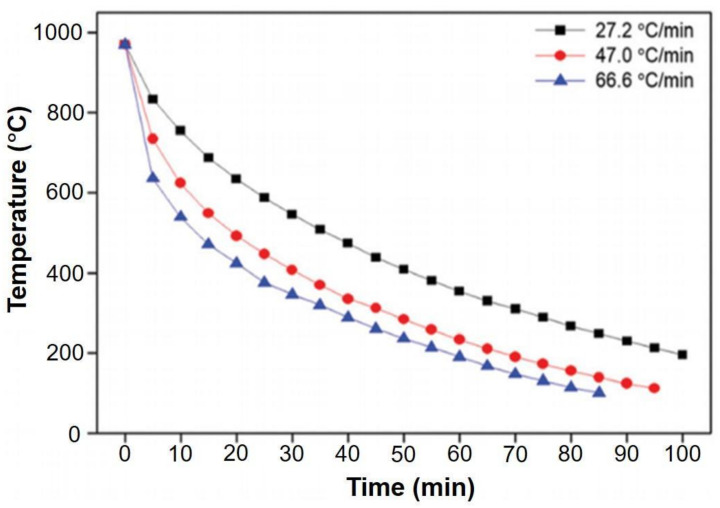
Cooling rate profiles of a CVD-MG. The initial cooling rates were estimated from the gradient between 0 and the first 5 min and were indicated in the upper-right part. Reprinted with permission from ref. [35].

**Figure 14:**
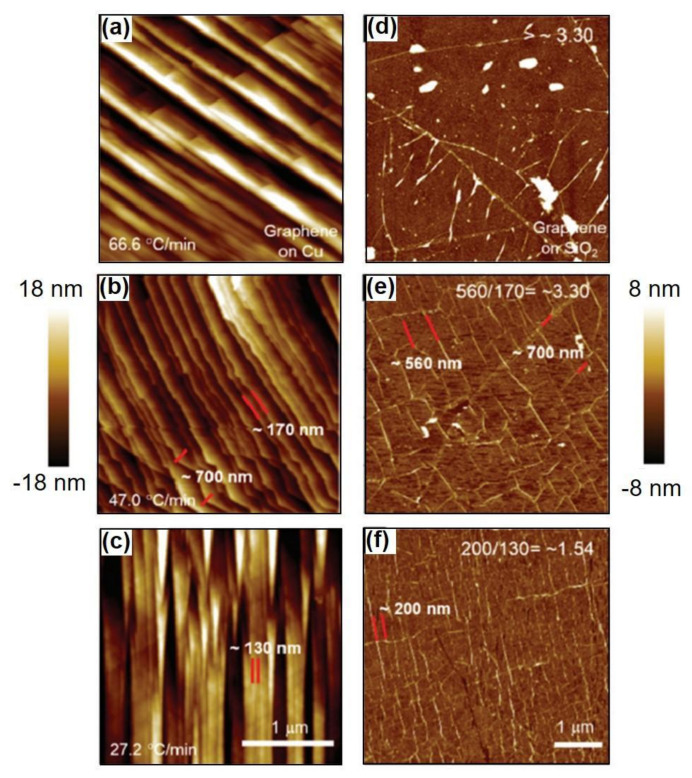
(**a**–**c**) Cu surface features were recorded by AFM studies subsequent to CVD-MG growth with varying rates of cooling (66.6, 47.0 and 27.2 °C min^−1^). (**d**–**f**) AFM images of CVD-MG subsequent to mounting on the SiO_2_/Si substrates. Characteristic distances between Cu nanoscale-terraces and RP-to-RP distances on SiO_2_/Si substrates were identified by the red bar. The values of expansion ratio obtained by dividing the RP-to-RP interval value on SiO_2_/Si substrates by the distances between Cu nanoscale-terraces were indicated in white. Reprinted with permission from ref. [35].

**Figure 15:**
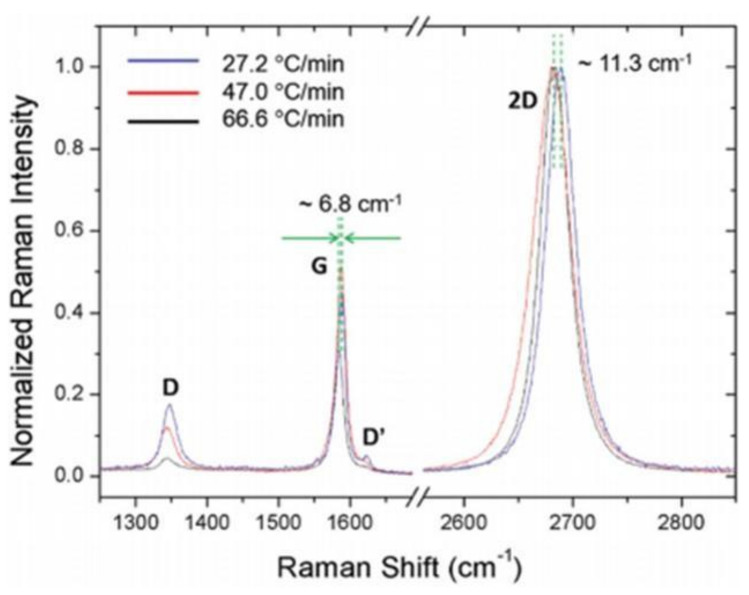
Representative Raman spectra were displayed for each cooling rate with graphene band assignments. The lowest cooling rate with blue spectrum and the highest cooling rate with black spectra of each G and 2D peak were indicated as red-shift from 27.2 °C/min to 66.6 °C/min of each G and 2D band was ~6.8 cm**^−^**^1^ and ~11.3 cm**^−^**^1^, respectively. Reprinted with permission from ref. [35].

Figure 13 showed the curve corresponding to the 3 different cooling rates, namely, 66.6, 47.0 and 27.2 °C min^−1^, utilized in this study. It is worthwhile to note that all the specimens were annealed at ~1000 °C and the rates of cooling were estimated from every curve during the initial 5 min and mentioned on the top-right region of Figure 13. Although this was not essentially a precise method to measure the rate of cooling, the properties of the CVD-MG exhibited evidently different tendencies with the estimated cooling rates. All the CVD-MG utilized in this study were grown and annealed at around 1000 °C, and the dissimilarity of the rate of cooling resulted in clear differences in morphology of the Cu foil surfaces and one of the CVD-MG subsequent to mounting onto the SiO_2_/Si substrates. Figure 14a–c showed the nanoscale-terrace shape on the surface of Cu substrate which exhibited evident dependence on the rate of cooling. It should be noted that the morphology (or the degree of corrugation) of the Cu surface should have shown at nearly the same degree in total 3 cooling rate cases since no variation in the growth conditions exclusive for the cooling rate was observed. Upon cooling, the expansion of CVD-MG due to a negative thermal expansion coefficient [63] occurred, whereas Cu contracted. If CVD-MG remains in contact with the surface of Cu substrate, there is a high possibility for the generation of compressive strain on CVD-MG after cooling due to the difference in the thermal expansion coefficients. Additional RPs would be generated if the strain was relieved by buckling of the CVD-MG. An attractive feature was revealed when the RPs on the mounted graphene films were examined as shown in Figure 14d–f. The numbers marked in Figure 14b–e and Figure 14c–f denote the distance between nanoscale-terraces on the Cu surface and the RP-to-RP distances of CVD-MG upon transfer, respectively, indicated by the red-bar. If the “expansion ratio” was defined as the ratio of the RP-to-RP distances to the one between the nanoscale-terraces on the Cu surface, this ratio, due to expansion, was greater than 1. Now, if three different cooling rates were compared to the expansion ratio, the clear trend could be unveiled. It was 1.54 for 27.2 °C/min and 3.30 for 47.0 °C/min. For 66.6 °C/min, the RP-to-RP distance was not clearly resolved, but the expansion ratio was definitely higher than 3.30. These results confirm that an efficient control on the cooling rate might be crucial to ascertain the morphology of CVD-MG before and after transfer. Figure 15 also showed the Raman spectra for the three different cooling rate cases subsequent to the transfer. Firstly, it could be compared to the introduction of sp^3^ defects (D′/D) for the individual cooling rates. This ratio was noticed to be the highest at the lowest cooling rate, and the lowest at the highest cooling rate. This behavior could be attributed to the association of some sp^3^-type defects with RPs. It should be noted that during the cooling process, the varying rates might influence re-crystallization of graphene which would influence the concentration of defects as well. However, it was difficult to differentiate the signals of such defects from those of morphological defects such as RPs. Secondly, focus should also be turned to the peak location of the G and 2D modes. A shift in both the bands to a lower frequency at a higher cooling rate was noticed. It was well-recognized that both doping and strain carry influence on the peak locations. The 2D band was less influenced by doping as compared to the G band [64,65,66], while the former was more sensitive to strain. The higher red-shift of the 2D band indicates its larger dependence on strain. The red-shifts of the G and 2D modes for the highest cool-down rate implied that certain compressive strain was relieved to the maximum possible extent at higher cooling rate.

**Figure 16:**
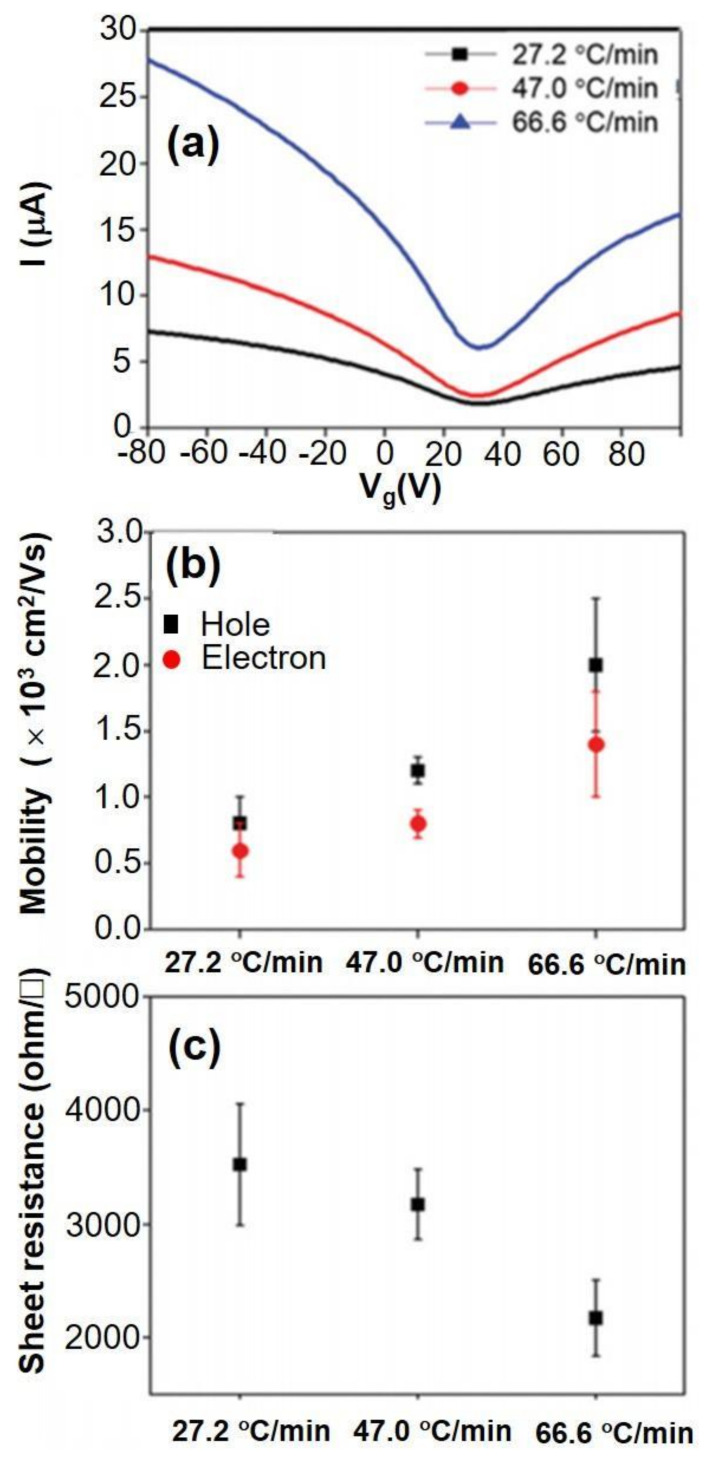
(**a**) Transferred I/V characteristic curves, (**b**) Carrier mobility and (**c**) Sheet resistance profile, at individual cooling rate. Reprinted with permission from ref. [35].

Figure 16a displayed the electrical characteristics of a graphene field effect transistor (GFET) as a function of the rate of cooling. A hydrophobic self-assembled monolayer (SAMs) with alkyl chains was sandwiched between the CVD-MG layer and the SiO_2_/Si substrate in order to avoid the substrate-induced doping effect. According to the results, GFET fabricated on SAMs with alkyl chains revealed increased charge carrier mobility with lower Dirac point voltages. The computed electron-hole mobility for each cooling rate was shown in Figure 16b. The cooling rate showed a direct relationship with electron-hole mobility values with almost the identical Dirac point voltages (~30 V). A similar trend was also noticed in the behavior of sheet resistance. However, the net absolute sheet resistance values were moderately high. The work should be focused on finding the trend in terms of precisely adjusting the detailed experimental conditions with changing RP densities and heights on the basis of tuning the cooling rate [35].

**Figure 17:**
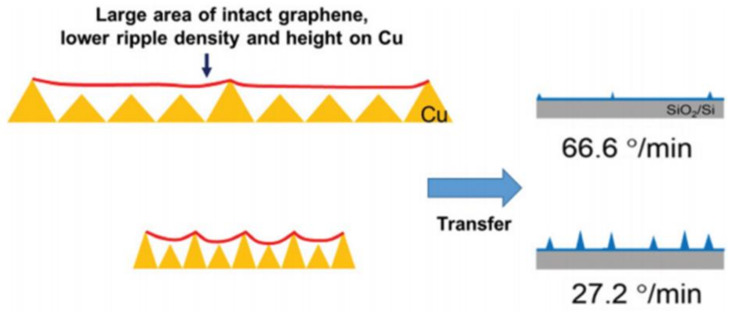
Overall schematic picture was shown by comparing two extreme cooling rates. Reprinted with permission from ref. [35].

Based on these observations, it could be claimed that the low RP densities and heights acquired by increased cooling rate comprised useful impacts in decreasing the flexural phonon scattering phenomenon, and thus resulted in higher lateral carrier mobility. A plausible mechanism for the increased electrical performances at higher cooling rate was visualized in Figure 17. At higher cooling rate, rough nanoscale-terrace formation would lead to lower RPs even if each nanoscale-terrace edge contributed to the formation of the RP. Nevertheless, it was investigated that at a higher rate of cooling, reduced portions of the nanoscale-terrace edges played an important role in forming the RPs, and further reduced their density. The fabricated configuration suggested that the CVD-MG was tauter at higher rates of cooling, which influenced the reduction in the height of the RP subsequent to transfer as shown in Figure 17. Thus, a precise method towards the nano-physical formation of CVD-MG implemented in this study by varying the rate of cooling might be one of the better approaches to fabricate electrically superior quality CVD-MG.

## 4. Conclusions

To summarize, in this review the characterization methods of CVD-MG are devised and developed for achieving a largescale, highly flexible, and transparent electrode. Various characterization techniques have been employed in this article to satisfy the overall purpose. Raman spectroscopy/microscopy could be efficiently utilized in order to unveil the direct contact effect between CVD-MG and Cu foil substrate in terms of suspending or supporting formation of CVD-MG. Macroscopically, Raman spectra between relatively even and uneven domains of CVD-MG on Cu foil substrate could be obtained. The relative tensile strain effects of G and 2D peak at uneven domain, which were relatively red-shifted, clearly indicated that CVD-MG was threaded and anticipated the presence of a suspended CVD-MG at uneven structure. Thus, the relatively large portions of suspended type CVD-MG played a pivotal role in enhancing lateral carrier mobility with reference to relatively less perturbation with underlying substrate (Cu foil or SiO_2_/Si). Considering z-directional suspended CVD-MG formation, an NPoM system could be devised such as Au NP/CVD-MG/Au film. It was an efficient platform in terms of using strongly enhanced incident z-polarized EM filed and resultant newly appeared (or enhanced) RBLM and other nano-scroll type (TA1, TA2, TA3) modes, called out-of-plane phonon vibrational modes of CVD-MG at the NPoM and it precisely characterized the detailed CVD-MG formation, especially, along the z-axis. Moreover, different shapes, positions, and intensities of out-of-plane vibrational modes were carefully examined via vectorial combination. It could be eventually claimed again that CVD-MG was not always flat and some domains heterogeneously protruded upward when probed by NPoM-SERS investigation. For the purpose of exploring in a nanoscale dimension, the EFM technique was employed. In particular, it could unravel and further substantiate the electrical performance of CVD-MG, in which the detailed surface structure of Cu foil substrate such as nano-valley or nano-peak was very crucial in terms of forming many suspended CVD-MG formations with resulting high tapping amplitude-based or wider phase-shifted electrostatic force mapping distributions. These results were in particular sync with the previous macroscopic Raman investigation. Dependence on ripple (RP) shape study using EFM and correlative Raman spectroscopy has been performed and the threading and surrounded type RPs showed different characteristics in which higher tapping amplitude could be observed at threading RPs. This indicated that structurally stable RPs showed relatively higher tapping amplitude, meaning that the threading type RP exhibited relatively hydrophobic or neutral compared to the surrounded. At this time, it could be additionally insisted that the shape effect of RP was the one of the important elements for determining higher electrical electrodes. Furthermore, the presence of RPs occurring during the fabrication process were very disturbed components in lateral carrier mobility flow on CVD-MG. Reducing the density and height of RPs has been tried. Rough nanoscale-terrace shapes at a higher rate of cooling would lead to lower RPs. In particular, it was noticed that for an increased rate of cooling, lesser fractions of the nanoscale-terrace edges actually contributed to the formation of RP which further reduced its density. Hence, the rate of cooling is a reliable parameter to control the electrical properties of CVD-MG. The introduction of the method in which the rate of cooling is tuned gives insight to forming largescale suspended graphene formations during the cooling step, which gradually leads to decreased RP densities and heights.

## Figures and Tables

**Table 1:** Spectral information from Figure 1 (Table 1 was reprinted with permission from ref. [6]).

	2D Peak Location (cm^−1^)	G Peak Location (cm^−1^)	G Peak FWHM (cm^−1^)
Dotted circle	2677.2	1588.9	30.5
Solid circle	2611.4	1602.1	26.4
